# The CsBES1-14-CsCOR413 module mediated by brassinolide positively regulates cold resistance in tea plant

**DOI:** 10.1093/hr/uhag098

**Published:** 2026-03-13

**Authors:** Chao Wang, Jinyu Yang, Yichen Zhao, De-Gang Zhao

**Affiliations:** Key Laboratory of Plant Resources Conservation and Germplasm Innovation in Mountainous Region (Ministry of Education), College of Life Sciences, College of Tea Sciences, Guizhou University, Guiyang, Guizhou 550025, China; Key Laboratory of Plant Resources Conservation and Germplasm Innovation in Mountainous Region (Ministry of Education), College of Life Sciences, College of Tea Sciences, Guizhou University, Guiyang, Guizhou 550025, China; Key Laboratory of Plant Resources Conservation and Germplasm Innovation in Mountainous Region (Ministry of Education), College of Life Sciences, College of Tea Sciences, Guizhou University, Guiyang, Guizhou 550025, China; Plant Conservation & Breeding Technology Center, Guizhou Key Laboratory of Agricultural Biotechnology, Guizhou Academy of Agricultural Sciences, Guiyang, Guizhou 550006, China

## Abstract

*Camellia sinensis* (L.) O. Kuntze exhibits severely restricted growth at low temperatures, resulting in reduced tea leaf yield and quality. BRI1-EMS-suppressor (BES) transcription factors, as key components of the brassinosteroids (BR) signaling pathway, are highly homologous to BZR and jointly regulate plants’ adaptation to environmental stress. In this study, the *CsBES1-14* gene was successfully cloned and identified from the transcriptome database of tea plant. Biochemical analyses identified *CsBES1-14* as a nuclear localized transcriptional activator, and BR and low temperature induced its expression. *Arabidopsis thaliana* plants overexpressing *CsBES1-14* exhibited increased chilling tolerance by promoting root growth and increasing the expression of cold responsive genes. Conversely, the suppression of *CsBES1-14* through virus-induced gene silencing (VIGS) in tea plant notably impaired cold tolerance. Transcription Factor-centered Yeast One-Hybrid screening identified *CsCOR413* as a downstream target, and electrophoretic mobility shift assays confirmed the direct binding of *CsBES1-14* to specific *cis*-elements in the *CsCOR413* promoter. Exogenous application of brassinazole (BRZ) and VIGS silencing experiments verified that the *ICE-CBF* cold response pathway could regulate the low-temperature-regulated protein CsCOR413. In summary, these findings elucidate that *CsCOR413* expression is modulated not only by the classic *ICE-CBF* signaling pathway but also directly regulated by *CsBES1-14*. These findings outline the key components of the cold resistance network in tea plant and provide novel molecular targets for genetic improvement strategies in perennial crops.

## Introduction

The tea plant (*Camellia sinensis* (L.) O. Kuntze) is a significant commercial crop, with its leaves serving as the raw material for producing three of the world’s most popular nonalcoholic beverages [1, 2]. Tea plants thrive in warm environments and have a low tolerance for cold temperatures [3]. Extended exposure of plants to subzero temperatures can harm cellular membranes, resulting in cell mortality [4, 5]. Therefore, low-temperature stress presents a considerable threat to tea yield and quality. Plants have evolved mechanisms and physiological changes to adapt to low temperature environments and to mitigate the damage caused by low temperatures [6, 7]. Additionally, plants enhance membrane stability through elevated unsaturated fatty acid content, thereby lowering lipid bilayer phase transition temperatures [8, 9]. At the same time, these organisms actively produce compounds like flavonoids, soluble sugars, and free proline (Pro) to minimize the harm inflicted by low temperatures [10, 11] and produce antifreeze proteins to enhance their resistance to low-temperature stress [12].

As endogenous chemical messengers, phytohormones orchestrate adaptive responses to external stimuli by modulating growth and developmental trajectories [13]. Under conditions of low temperature, plant hormone levels consistently vary, triggering genes linked to the signaling pathways of plant hormones to engage in the response to cold stress [14–16]. Plant hormones, including abscisic acid (ABA), salicylic acid (SA), methyl jasmonate (MeJA), and BR, function as key regulators of plant responses to cold stress [17–19]. For example, SA can alleviate oxidative stress caused by low temperatures by enhancing the function of antioxidant enzymes and decreasing levels of malondialdehyde (MDA), thereby increasing the stability of plant cell membranes [20]. The exogenous application of ABA and MeJA can considerably enhance the cold resistance of plants [21]. Administration of exogenous BR upregulates key genes in the BR pathway. Additionally, BR signaling influences the expression levels of various genes through the crucial transcription factor BRI1-EMS-SUPPRESSOR/BRASSINAZOLE RESISTANT (BES/BZR) found in the BR signaling pathway. This mechanism alleviates cold-induced damage in plants and supports their adaptation to environmental stress [22, 23]. The BES/BZR family members are crucial for how plants respond to stress caused by low temperatures [24–26]. Spraying different concentrations of 2,4-epibrassinolide can enhance the cold resistance of cotton, with high concentrations showing more pronounced effects [27]. The increased expression of BRASSINOSTEROID INSENSITIVE 1 (*BRI1*) result in diminished cold tolerance in plants, while both the *bri-9* mutant and the overexpression of *BRI1* can elevate the expression levels of *CBF* and *COR* when subjected to normal temperature conditions [28]. In tomato, *BZR1* directly interacts with the *CBF1* promoter region. By crosstalking with ABA signaling, *BZR1* elevates *NCED1* transcript levels, leading to CBF upregulation and consequent frost tolerance augmentation [29].

The ICE1-CBF-COR transcriptional cascade, which relies on *CBF* signaling, is a typical cold response signal regulatory pathway [30, 31]. In tea plant, the hexokinase gene *CsHXK4* enhances cold resistance through the *CBF* signaling pathway [32]. Oppositely in tomato systems, *SlMPK1*-*SlMPK2* heterodimers bind phosphorylated *SlBBX17*, facilitating intensified *SlBBX17–SlHY5* interactions. Cold exposure amplifies *SlHY5*’s transcriptional regulation of *SlCBF* via this process, elevating tomato chilling tolerance [33]. Strigolactones boost plant cold tolerance by alleviating *WRKY41*-mediated suppression of *CBF/DREB1* expression [34]. Furthermore, numerous genes exhibit responses to low temperatures that are not dependent on the CBF signaling pathway. In tea plant, *CsCIPK20* competes with *CsVTC1* for binding to *CsCSN5*, which safeguards *CsVTC1* from being degraded by *CsCSN5*, thus promoting ascorbic acid (AsA) accumulation and bolstering cold tolerance [35]. Moreover, the enzyme betaine aldehyde dehydrogenase (*CsBADH1*) enhances cold tolerance in *C. sinensis* through modulating the glycosylation process of apigenin [17]. *CsGAT1* manages the capacity of tea plant to endure low-temperature stress by adjusting both intracellular and extracellular levels of γ-aminobutyric acid (GABA) [36] .

This study focuses on the BES1/BZR1 family of transcription factors, which act as key transcriptional effectors of the BR signaling pathway and are known to regulate diverse stress responses in model plants. However, their specific functions in tea plant remain largely unexplored. Transcriptomic profiling of cold-challenged tea plant demonstrated strong *CsBES1-14* induction in response to low temperatures. Following BR application, *CsBES1-14* gene expression was significantly higher in cold-stressed plants relative to non-BR-treated counterparts. In this study, to assess the functional impact of *CsBES1-14* under cold stress, it was overexpressed in both wild-type (WT) *Arabidopsis thaliana* and the *bes1-1* loss-of-function mutant, and knocked down in tea plant. Comprehensive identification of *CsBES1-14*-interacting genes enabled mechanistic dissection of cold stress adaptation, facilitating molecular breeding strategies in tea plant.

## Results

### The expression pattern and protein localization of the *CsBES1-14* gene

**Figure 1:**
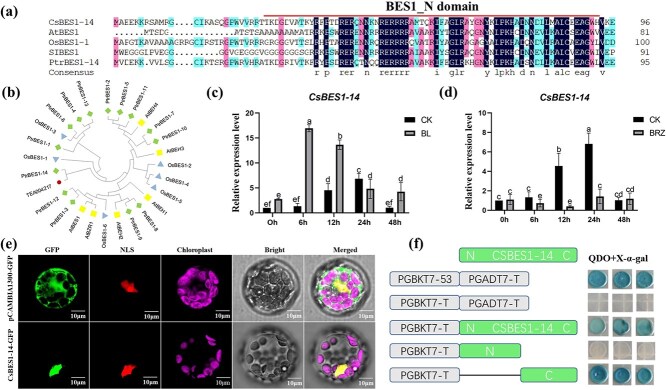
The expression pattern and protein localization of the *CsBES1-14* gene. (a) Multisequence alignment of BES in different plants. (b) Evolutionary tree analysis. (c) The transcriptional level of *CsBES1-14* in tea plant after cold treatment with BL. (d) The transcriptional level of *CsBES1-14* in tea plant after cold treatment with BRZ. (e) Cellular localization of *CsBES1-14* in *A. thaliana* protoplasts: controlled by GFP empty vector; *CsBES1-14*-GFP: green fluorescence channel of the fusion protein of the target gene and GFP; Chloroplast: autofluorescence red channel. (f) Verification of transcriptional activation activity of pGBKT7-*CsBES1-14.*

Analysis of transcriptome data from cold-stressed tea plant demonstrated notable upregulation of the TEA004217 gene. The TEA004217 gene encodes a 175-amino acid protein, defined as an unstable hydrophilic protein. To clarify the functional and evolutionary connections of TEA004217, a phylogenetic tree was created by utilizing protein sequences from six homologs of BES1/BZR1 in *A. thaliana*, six BES1 homologs in *Oryza sativa*, eight BES1 homologs in *Solanum lycopersicum*, and 14 BES1 homologs in *Populus trichocarpa*. Phylogenetic analysis indicates that the TEA004217 protein in tea trees is closely related to the PtrBES1-14 protein in poplar trees, suggesting that there may be functional similarities between the two (Fig. 1b). Therefore, it was named *CsBES1-14*. Alignment of the amino acid sequences revealed that the CsBES1-14 protein featured a BES1_N domain that is strongly conserved, thereby categorizing it within the *BES/BZR* gene family (Fig. 1a).

Transcript profiling demonstrated brassinolide (BL)-induced upregulation of *CsBES1-14*, with significant expression enhancement following exogenous BL application (Fig. S1a). In contrast to the control group that did not undergo BR treatment, tea plant subjected to BR exhibited a significant increase in the expression of the *CsBES1-14* gene when facing cold stress (Fig. 1c). In contrast, brassinazole (BRZ) induction led to a notable decrease in its expression, implying that *CsBES1-14* plays a role in cold resistance mediated by BR (Fig. 1d). Further analysis identified low-temperature-responsive elements within the *CsBES1-14* promoter. A recombinant vector harboring the *CsBES1-14* promoter driving *GUS* expression was created. *GUS* staining demonstrated that low temperatures enhanced the activity of the *CsBES1-14* promoter, a finding further corroborated by Reverse Transcription Quantitative PCR (RT-qPCR)(Fig. S1b). This implicates *CsBES1-14* as a key regulator of cold stress responses (Fig. S1e). In order to clarify the molecular role of *CsBES1-14*, the expression patterns specific to various tissues of *CsBES1-14* in tea plant were examined. *GUS* histochemical staining of transgenic plants confirmed high expression of *CsBES1-14* in leaves and roots but low expression levels in stems and flowers (Fig. S1c and d). Analysis of CsBES1-14 subcellular localization has shown its main site of accumulation to be the nucleus, and the C-terminus was identified as the domain responsible for transcriptional activation (Fig. 1e and f).

### Heterologous expression of *CsBES1-14* confers cold tolerance to *A. thaliana* by regulating its cold signaling pathway

**Figure 2:**
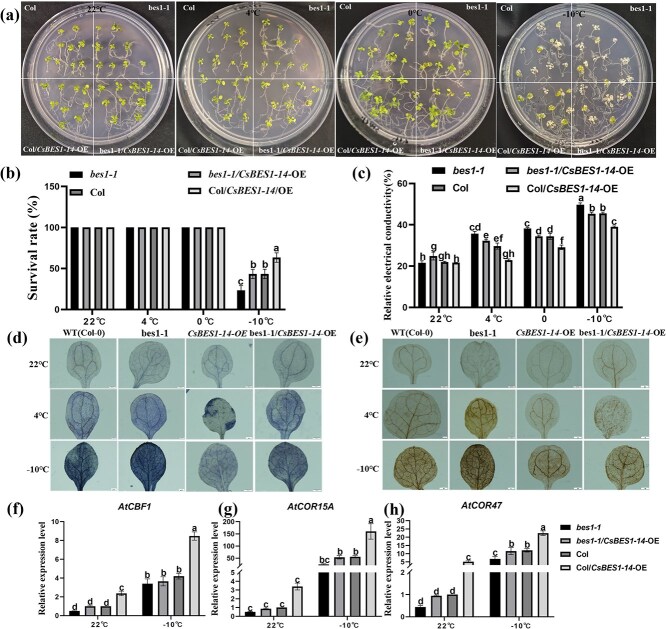
Allogeneic expression of *CsBES1-14* enhances cold resistance in *A. thaliana*. (a) Growth status of *A. thaliana* with different genotypes at various temperatures. (b) Survival rates of different genotypes of *A. thaliana* after exposure to various temperature treatments. (c) Relative conductivity of *A. thaliana* after different temperature treatments. (d) DAB histochemical staining. (e) NBT histochemical staining. (f)–(h) Transcript profiling of cold responsive genes *AtCBF1*, *AtCOR15A*, and *AtCOR47*. Note: Col: Columbia ecotype WT *A. thaliana*; *bes1-1*: *BES1* Deficiency Mutant; Col/*CsBES1-14*-OE: *CsBES1-14* Transgenic Columbia Ecotype; *bes1-1/CsBES1-14-OE*: *CsBES1-14*-Transformed *bes1-1* Mutant.

The impact of *CsBES1-14* on *A. thaliana* cold tolerance was investigated by subjecting Col-WT, *bes1-1* mutants, Col/*CsBES1-14*-OE complementation lines, and *bes1-1*/*CsBES1-14*-OE complementation lines to cold stress. Compared to the WT, the *CsBES1-14-*overexpressing *A. thaliana* exhibited stronger cold resistance under freezing stress, with a significantly higher survival rate (Fig. 2a). Additionally, the survival rate of the *bes1-1* mutant *A. thaliana* increased to 44% after genetic complementation, which is close to that of the WT (Fig. 2b). Concurrently, the leakage of electrolytes indicates the extent of cellular membrane damage in plants under environmental stresses. The analysis of *A. thaliana* plants treated at different temperatures showed that the relative conductivity of *CsBES1-14*-overexpressing *A. thaliana*, WT *A. thaliana*, and *bes1-1* mutant *A. thaliana* increased continuously as temperature decreased. The rise in conductivity for plants overexpressing *CsBES1-14* was significantly less than that observed in WT *A. thaliana* (Fig. 2c). Additionally, we examined reactive oxygen species (ROS) in *A. thaliana* leaves under cold stress. NBT and DAB staining indicated that *CsBES1-14* reduced the accumulation of ROS (Fig. 2d and e). The findings indicate that the increased expression of *CsBES1-14* could influence the buildup of ROS and the loss of electrolytes during cold stress, thereby enhancing the plant’s tolerance to cold conditions. Physiological analysis revealed that, compared to the control, the *CsBES1-14*-overexpressing lines exhibited a significant decrease in Pro content and peroxidase (POD) activity, whereas the activities of catalase (CAT) and superoxide dismutase (SOD) were markedly increased. The complemented line showed no significant differences from the WT *A. thaliana*. These findings collectively indicate that *CsBES1-14* enhances cold tolerance in *A. thaliana* (Fig. S2).

Recognizing the CBF–COR cascade’s importance for cold tolerance, we quantified the transcript abundance of *CBF* genes and their downstream effectors in the transgenic lines. Transcript abundance of key cold-responsive genes (*AtCBF1*, *AtCOR15A*, *AtCOR47*) was quantified. Transcriptional upregulation of *AtCBF1*, *AtCOR15A*, and *AtCOR47* following *CsBES1-14* overexpression implicates this regulator in cold stress modulation via the ICE–CBF–COR signaling cascade (Fig. 2f–h).

### The effect of inhibiting the *CsBES1-14* gene on tea plants’ ability to withstand cold temperatures

**Figure 3:**
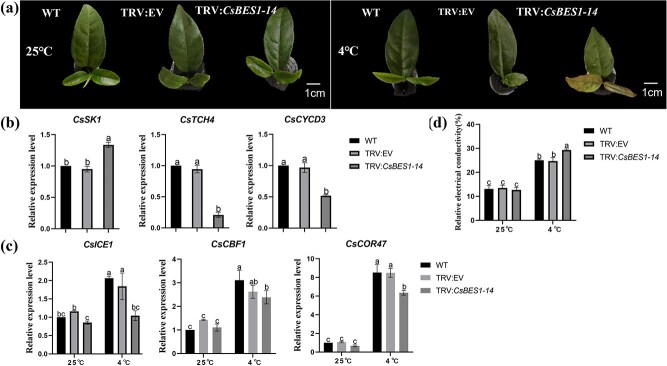
The impact of *CsBES1-14* silencing on the cold tolerance of tea plant is significant. (a) Examination of the phenotypic traits related to the cold tolerance in various tea plant varieties. Note: normal temperature control; Growth of tea plant treated at 4°C. (b) Expression profiling of *CsSK1*, *CsTCH4*, and *CsCYCD3*, components of the BR signaling pathway. (c) Transcript abundance of *CsICE1*, *CsCBF1*, and *CsCOR47* in cold-stressed tea plant. (d) The analysis of electrical conductivity for the tea leaves of WT, TRV: EV, and TRV: *CsBES1-14* was conducted at different temperature ranges. TRV: EV: Empty Vector; TRV: *CsBES1-14*: Plants in which the *CsBES1-14* was silenced.

The functional role of *CsBES1-14* in tea plant cold tolerance was confirmed through virus-induced gene silencing (VIGS) assays, as evidenced by the significant suppression of its expression in the tobacco rattle virus (TRV):*CsBES1-14* knockdown lines (Fig. S3a and b). Following this, tea plant that had undergone *CsBES1-14* silencing were exposed to cold conditions. While leaves from the WT and TRV: EV groups returned to their normal state after being placed at room temperature, the leaves from the TRV: *CsBES1-14* group displayed signs of frost damage and yellowing (Fig. 3a). Assessments of electrolyte leakage after cold exposure indicated a statistically significant increase in the TRV: *CsBES1-14* line versus the WT and TRV: EV controls. Physiological assessment revealed that silencing *CsBES1-14* in tea plant triggered a significant increase in Pro content and POD activity, coupled with a marked decrease in CAT and SOD activities compared to the control. These collective responses further confirm its positive role in enhancing cold tolerance (Fig. S4). These findings demonstrate that silencing *CsBES1-14* compromises tea leaf cell membrane integrity under cold stress, leading to greater damage (Fig. 3d). Following transient overexpression of *CsBES1-14* in tea plant, the transformed leaves displayed substantially greater chilling resistance relative to the control, which was accompanied by a significant decrease in relative electrolyte leakage (Fig. S5). Furthermore, the impact of *CsBES1-14* knockdown on transcript abundance of the *CsICE1*, *CsCOR*, and *CsCBF1* genes was assessed in *C. sinensis* foliage. Every sample exposed to cold conditions at 4°C demonstrated a notable reduction in the expression of *CsCBF1*, *CsICE1*, and *CsCOR47*, with the treatment group displaying the most marked decline (Fig. 3c). The impact of *CsBES1-14* silencing on BR signaling pathway gene expression was also assessed. Postsilencing of *CsBES1-14*, there was a significant increase in the expression of *CsSK1*, while *CsTCH4* and *CsCYCD3* expressions significantly reduced (Fig. 3b), indicating that the lack of *CsBES1-14* impaired BR signal transduction. These results demonstrate that suppressing *CsBES1-14* impairs BR signaling, thereby directly impacting cold stress responses and reducing shoot cold tolerance in tea plant.

### The expression of *CsCOR413* is activated transcriptionally by *CsBES1-14*

**Figure 4:**
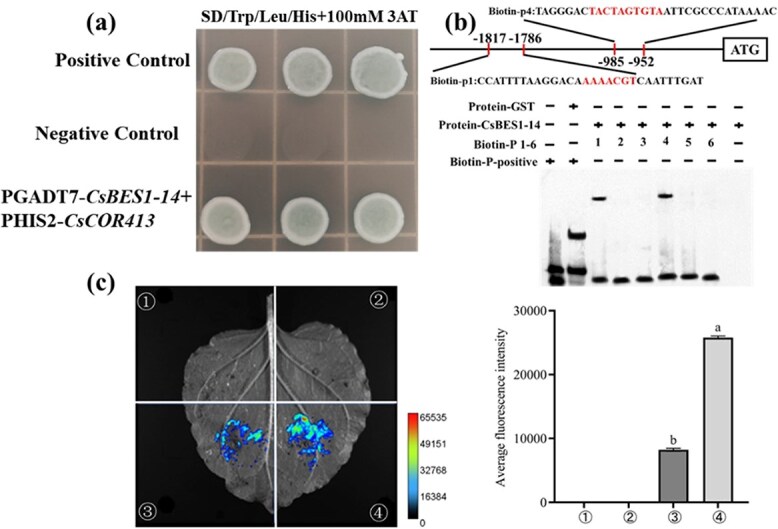
Interaction verification between *CsBES1-14* and *CsCOR413* promoter. (a) Yeast single-hybrid verification interaction; Positive Control: pHIS2-p53 + pGAD53m; Negative: pGADT7-*CsBES1-14* + pHIS-p53; pGADT7 + pHIS2-*CsCOR413*: *CsCOR413* promoter self-activation; pGADT7-*CsBES1-14* + pHIS2-*CsCOR413*: experimental group. (b) EMSA showed the binding activity of *CsBES1-14*-GST and the *CsCOR413* promoter. Probes labeled with biotin were incubated alongside CsBES1-14-GST, while acrylamide gels facilitated the separation of free DNA from that which was bound. An equal amount of CsBES1-14-GST was added to each lane. (c) Interaction between *CsBES1-14* and *CsCOR413* promoter double luciferase. ① 0800-LUC + 62-SK; ② 62-SK-*CsBES1-14*; ③ 0800-LUC-*CsCOR413*; ④ 0800-LUC-*CsCOR413* + 62-SK-*CsBES1-14*.

To further delineate the regulatory network of *CsBES1-14* under cold stress, a yeast reverse one-hybrid assay was used to screen the downstream target genes. Six proteins associated with cold stress were identified, among which cold-responsive proteins (COR) are a class of proteins induced by cold and are involved in regulating plant cold resistance. It has been hypothesized that COR proteins are involved in the response of the *CsBES1-14* gene to cold stress. Therefore, *CsCOR413* (TEA000894.1), which encodes the *COR* gene in tea plant, was selected for cloning and subsequent studies. To investigate *CsBES1-14*’s direct regulation of *CsCOR413*, we conducted yeast one-hybrid (Y1H) assays. Binding of *CsBES1-14* to the *CsCOR413* promoter was confirmed by growth of cotransformed yeast colonies on selective media (Fig. 4a). Subsequent dual-luciferase assays in tobacco coinfiltrated with 0800-LUC-*CsCOR413* and 62-SK-*CsBES1-14* showed significantly increased LUC activity compared to that of the control group (Fig. 4c). Additionally, electrophoretic mobility shift assay (EMSA) corroborated the findings of the Y1H and dual-luciferase assays, confirming that *CsBES1-14* promoted the transcriptional activity of *CsCOR413* by binding to its specific binding sites AAAACGT and TACTAGTGTA in the promoter region (Fig. 4b).

### The cold-associated protein CsCOR413 enhances cold tolerance in tea plant

**Figure 5:**
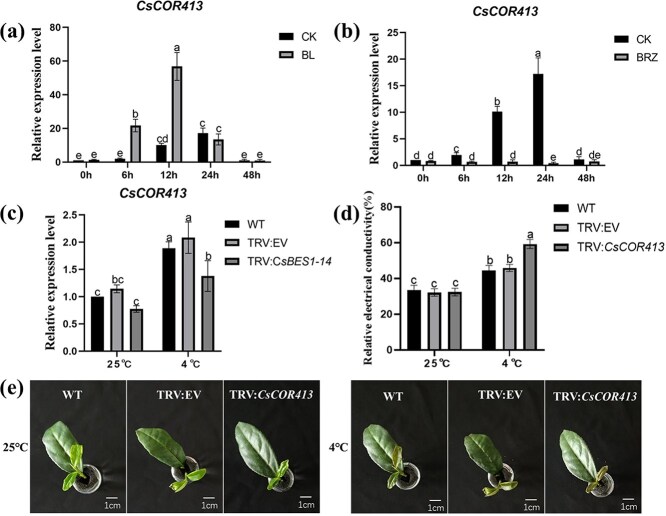
Expression pattern of *CsCOR413* and analysis of cold resistance in tea plant following its silencing. (a) Transcriptional levels of *CsCOR413* following cold treatment of tea plant after BL application. (b) Transcriptional levels of *CsCOR413* following cold treatment of tea plant after BRZ application. (c) Analysis of *CsCOR413* expression in silenced plants. (d) Analysis of leaf relative conductivity of different plants under cold stress. (e) Phenotypic analysis of plants silenced by *CsCOR413* under low-temperature stress.

Taking into account that *CsCOR413* serves as a downstream target of *CsBES1-14*, we proposed that *CsBES1-14* could influence BR signaling transduction during cold stress through the direct modulation of *CsCOR413*. Subsequent analysis through RT-qPCR confirmed that BR, BRZ, and cold stress triggered the expression of *CsCOR413* (Fig. 5a and b). To explore this hypothesis, we examined the expression levels of *CsCOR413* in both WT and *CsBES1-14*-silent lines, finding a significant reduction in *CsCOR413* expression (Fig. 5c). To delineate the role of *CsCOR413* in cold stress responses, *CsCOR413*-knockdown tea plant were challenged with a 4°C regime. Cold stress symptoms were observed in both WT and TRV: EV plants; however, normal growth resumed upon restoration of standard growth temperatures. Conversely, TRV: *CsCOR413* lines exhibited pronounced foliar necrosis and heightened cold susceptibility, indicating impaired freezing tolerance due to *CsCOR413* knockdown (Fig. 5e). Moreover, we measured the relative electrical conductivity of WT, TRV: EV, and TRV: *CsCOR413* tea plant leaves. Following the cold treatment, plants with silenced *CsCOR413* exhibited a notable increase in relative conductivity when compared to control plants, indicating enhanced membrane permeability and reduced cold tolerance (Fig. 5d). Assessment of physiological parameters in the *CsCOR413*-silenced line under low temperature indicated that *CsCOR413* silencing led to higher Pro accumulation and POD activity along with suppressed CAT and SOD activities. This pattern suggests an elevated state of oxidative stress and a compromised antioxidant capacity. (Fig. S6). Transient expression of *CsCOR413* in tea leaves revealed that, compared to the control group, leaves overexpressing this gene exhibited a significant enhancement in cold tolerance and a marked reduction in relative electrical conductivity (Fig. S7). Transgenic *A. thaliana* lines overexpressing *CsCOR413* (T3 generation) showed substantially improved performance under cold stress, including less visible injury and significantly decreased electrolyte leakage compared to nontransgenic plants. This results further demonstrate that *CsCOR413* plays a positive regulatory role in plant cold resistance (Fig. S8). Importantly, several studies have indicated that the *COR* gene is regulated through the traditional cold pathway involving ICE–CBF. To confirm this, the expression of *CsCOR413* was assessed within WT and *CsICE1-*silencing contexts, and a significant decrease in its expression levels was noted. (Fig. S9a–c). These findings collectively suggest that *CsCOR413* expression is modulated by the ICE–CBF cold-signaling pathway and is also directly influenced by the crucial factor *CsBES1-14* within the BR signaling pathway, uncovering a novel mechanism of interpathway regulation within the cold tolerance framework of tea plant.

## Discussion

BES/BZR transcription factors critically regulate brassinosteroid-mediated abiotic stress responses throughout diverse plant species [24]. Although the BR signaling pathway has been well characterized in model plants, its molecular mechanisms remain undefined in tea plant, particularly with regard to cold stress adaptation [24, 36, 37]. *CsBES1-14* was identified as a BR-responsive BES/BZR transcription factor regulating cold stress adaptation through systematic screening of early cold stress-induced transcriptomic profiles in tea plant.

Gene expression patterns provide important insights for investigating biological functions [36]. Promoters of *CsBZR1* genes in both wild and cultivated tea plant harbor cold-responsive *cis*-elements, with *lj43CsBZR1s* and *sczCsBZR1s* displaying pronounced transcriptional activation under chilling stress [1]. This functional conservation is consistent with the cross-species characteristics of *BES/BZR* gene family members. For example, homologous gene promoters in *Nicotiana benthamiana*, *Eucalyptus*, and *Brassica oleracea* contain low-temperature response elements and exhibit specific cold responses [38–40]. This research demonstrated that the promoter of the *CsBES1-14* gene includes regulatory elements that respond to low temperatures through cloning and analysis. Further investigation using p1391Z-*CsBES1-14*-GUS transgenic *A. thaliana* confirmed the promoter activity. Cold-stressed transgenic plants showed amplified *GUS* transcript levels in roots/leaves and concurrent potentiation of staining intensity. This finding provides clear evidence for the essential role of *CsBES1-14* in how tea plant responds to cold stress. Moreover, following BR treatment and cold stress, *CsBES1-14* gene expression in tea plant peaked at 6 h. This is because the BR signaling pathway is activated by BL treatment in the early stage, enabling related proteins to respond relatively quickly, thereby reducing cold damage to plants. It is speculated that *CsBES1-14* participates in cold stress regulation via BR.

BES/BZR genes positively regulate cold resistance in plants [37, 41]. In cotton, compared to WT, *A. thaliana*-overexpressing *GhBZR15* exhibits enhanced cold tolerance [42]. By blocking sucrose degradation pathways, *PpBZR1* overexpression elevates freezing tolerance in *A. thaliana*, manifesting as increased germination capacity and post-chilling survival [38]. *BZR1* strengthens cold tolerance in *A. thaliana* by promoting the expression of *CBF*, *WRKY6,* and *PYL6* [18]. Low-temperature stress assays revealed that *CsBES1-14*-overexpressing *A. thaliana* lines showed significantly increased survival rates coupled with reduced leaf relative electrical conductivity and suppressed ROS accumulation, corroborating previous studies [43]. Because the *CsBES1-14* promoter exhibits tissue specificity, it was significantly expressed in the roots/ leaves. To verify *CsBES1-14*’s function, we silenced this gene in tea leaves via VIGS and exposed silenced plants to low temperatures. The results of tea plant leaves silenced with *CsBES1-14* were consistent with those of previous studies. The pronounced yellowing observed in TRV: *CsBES1-14* plants, but not in controls, under cold stress suggests that silencing *CsBES1-14* may impair chloroplast function or energy homeostasis, rendering plants more susceptible to chilling injury. Although VIGS can potentially introduce nonspecific stress, the consistent results from rigorous empty-vector controls and multiple physiological assays in this study support the conclusion that the yellowing phenotype stems primarily from the loss of *CsBES1-14* function and represents an integral component of enhanced cold sensitivity. The tissues with *CsBES1-14* silenced exhibited heightened cold sensitivity and markedly elevated electrolyte leakage, confirming that *CsBES1-14* loss amplifies plant susceptibility to chilling stress. Notably, in the TRV: *CsBES1-14* knockdown lines, the expression of cold-responsive genes *CBF1* and *COR47* was only slightly suppressed, unlike the significant upregulation observed in *CsBES1-14* overexpression *A. thaliana* lines. It is therefore hypothesized that *CsBES1-14* may be involved in only one specific branch of the cold tolerance pathway. When *CsBES1-14* is silenced, other cold resistance pathways may remain functional, thereby partially compensating for the loss of its function under cold stress. In addition to directly or indirectly activating downstream cold-responsive genes, *CsBES1-14* also coordinates the cold adaptation process in tea plant at multiple levels by integrating the BR signaling pathway. This role is supported by the coordinated expression shift of *CsSK1*, *CsTCH4*, and *CsCYCD3* upon its silencing (Fig. 3B), indicating that *CsBES1-14* modulates a suite of processes ranging from upstream signal relay and rapid cell wall remodeling to cell cycle progression. Such multifaceted regulation suggests that BR signaling mediated by *CsBES1-14* not only initiates specific defense programs but also fine-tunes overall growth and development, likely redirecting resources toward stress survival and thereby establishing an integrated mechanism that enhances chilling tolerance. In addition, in both *CsBES1-14-*silenced and overexpressed plants, the activities of antioxidant enzymes (SOD, CAT, and POD) and the content of Pro, which are key physiological indicators for evaluating the cold tolerance of plants to low-temperature stress [43, 44], all showed coordinated and corresponding changes. Meanwhile, the overexpression lines exhibited a markedly enhanced ability to clear ROS, as evidenced by ROS staining, which showed significantly lower accumulation of H₂O₂ and O₂^−^ compared to controls. These results demonstrate that *CsBES1-14* plays an important role in plant cold tolerance by positively regulating antioxidant defense and osmotic adjustment, thereby improving cellular redox homeostasis under chilling stress. In conclusion, *CsBES1-14* plays a role in managing cold responses and is likely to enhance the cold resistance of plants.

Among the 4000–5000 genes regulated by BR, ~2000 are regulated by BES/BZR [36, 45, 46]. The BES/BZR transcription factors not only regulate plant growth and development but also function to enhance stress tolerance by interacting with other transcription factors [47, 48]. The transcription factor *SlBZR1D* confers enhanced salt tolerance to tomato by positively regulating the expression of participating genes such as *SlAPX2*, *SlAREB1*, *SlCAT2*, *SlRD29*, *SlERF1*, and *SlDREB1* [31]. The relationship between *WRKY54* and *BES1* contributes to the drought response in *A. thaliana* [49]. To pinpoint *CsBES1-14-*interacting proteins under cold stress, TF-centered Y1H screening was conducted, revealing *CsCOR413* as a cold-regulated candidate. Related studies have indicated that *COR* (cold-regulated) genes encode proteins involved in osmotic adjustment and cold tolerance [50]. Studies indicate that members of the COR protein family play diverse roles in plant cold adaptation, often involving processes such as membrane stabilization, metabolic regulation, and signal transduction. For instance, the chloroplast inner membrane-localized COR413 protein (COR413im) is considered a metabolic transporter crucial for maintaining chloroplast homeostasis and metabolic balance under complex environmental conditions; its function is directly linked to membrane system stability under stress [51]. In contrast, the chloroplast stroma-localized COR15A protein can participate in cold response by forming protective oligomers to prevent the inactivation of key enzymes during freezing or by directly binding to potential substrates [52]. Furthermore, overexpression of *PsCOR413im1* has been shown to upregulate the expression of cold-responsive genes such as *AtCOR15* in *A. thaliana*, thereby synergistically enhancing cold adaptation [53]. In particular, the *COR413pm* gene enhances Ca^2+^ influx and upregulates the expression of stress responsive *COR* genes in *A. thaliana*, thereby contributing to the regulation of cold stress responses [53]. Y1H, dual-luciferase reporter, and EMSA assays confirmed direct binding of *CsBES1-14* to *cis*-elements AAAACGT/TACTAGTGTA in the *CsCOR413* promoter, dramatically enhancing its transcription. Moreover, the examination of the expression pattern for the *CsCOR413* gene indicated that after BL spraying and subsequent cold stress treatment, the expression trend of *CsCOR413* aligned with that of *CsBES1-14*, showing a gradual increase over time and reaching its highest level at 6 h. BRZ-mediated inhibition of BR signaling prior to cold stress resulted in significantly reduced *CsBES1-14* transcript levels in treated versus control plants. Simultaneously, the expression level of *CsCOR413* also displayed a similar trend of downregulation. This further verified that *CsBES1-14* directly regulates *CsCOR413*. At the same time, the expression level of *CsCOR413* also displayed a similar trend of downregulation significant increase in cold sensitivity. The ICE–CBF regulatory network in the classic plant cold-resistance pathway plays a crucial role in tea plant [54]. To verify whether COR is regulated by the ICE1–CBF signaling pathway, specific suppression of *CsICE1* expression was achieved using VIGS technology. The findings indicated a notable reduction in the transcriptional level of *CsCOR413*. *BZR1* modulates CBF-independent cold stress genes—including *ESM1*, *WRKY6*, *JMT*, *SOC1*, *SAG21*, and *PYL6*—in *A. thaliana*, thereby mediating unique cold-response mechanisms [18]. These experimental results collectively demonstrate that the expression of the cold-regulated protein *CsCOR413* is co-regulated by the classic ICE–CBF signaling pathway and directly modulated by *CsBES1-14*, a key factor in the BR signaling pathway. This demonstrates a new perspective on cross-pathway regulation within the cold tolerance network of tea plant.

Collectively, our evidence indicates that *CsBES1-14* functions as a positive cold stress regulator, conferring enhanced low-temperature tolerance to tea plant through the direct or indirect upregulation of *CsCOR413*. This research offers initial understanding of the mechanisms regulating cold tolerance in tea plant, presenting fresh perspectives and methods for the genetic improvement of these plants.

## Conclusion

**Figure 6:**
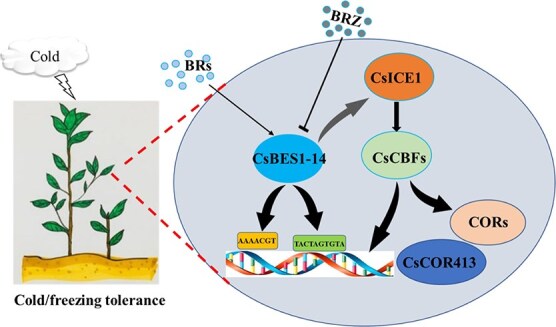
Regulatory framework for *CsBES1-14* and *CsCOR413* in mediating tea plant cold tolerance.

Through comprehensive analysis, it was determined that CsBES1-14, a key transcription factor in BR signaling, enhances cold tolerance in tea plant by regulating cold stress responses. Mechanistic investigations demonstrated that *CsBES1-14* enhances cold resistance via the BR signaling pathway-mediated transcriptional activation of the cold-inducible protein *CsCOR413*. Additionally, *CsCOR413* expression was coordinately regulated by the canonical *ICE–CBF* signaling pathway, revealing a cross-regulatory interplay between distinct cold adaptation networks (Fig. 6).

## Materials and methods

### Plant materials

Genetic transformation was carried out using the Colombian WT (Col-0) and the loss-of-function mutant *A. thaliana bes1-1* (SALK_098634) in the experiment. Seeds of the Col WT *A. thaliana* were maintained and propagated internally by our research group. The *bes1-1* mutant was obtained from the Arashare platform. *Camellia sinensis* ‘Qiancha 1’was cultivated in climate-controlled chambers at Guizhou University’s Key Laboratory for Conservation of Mountainous Plant Resources and Germplasm Innovation (Ministry of Education).

### Bioinformatics analyses of *CsBES1-14*

Retrieve BES/BZR family protein sequences for *A. thaliana* (https://www.arabidopsis.org/), *O. sativa* (https://plants.ensembl.org/index.html), and *P. trichocarpa* (https://phytozome-next.jgi.doe.gov/) from their respective databases. A neighbor-joining phylogenetic tree was created utilizing MEGA-11 (https://www.megasoftware.net/) software. Additionally, employ DNAMAN (https://www.dnaman.net/) software to further analyze and enhance the results of the multiple sequence alignment.

### Strains and vectors

GV3101 (*Agrobacterium tumefaciens*) and DH5α (*Escherichia coli*) strains were routinely maintained in the laboratory. The pCAMBIA1391Z vector was designed and held by our laboratory. Vectors including pMD®18-T Vector, pCAMBIA1300-35S-GFP, pTRV1, and pTRV2 were obtained from Wuhan TransGen Biotech.

### Subcellular localization

The subcellular localization of CsBES1-14 was analyzed using a vector constructed based on pCAMBIA1300-35S-GFP. Protoplasts were isolated from the leaves of WT *A. thaliana* (at the eight-leaf stage) grown under normal conditions, using an *A. thaliana* Protoplast Isolation and Transformation Kit (Coolaber, Cat#: PPT101-10T). The constructed plasmid was then transfected into the protoplasts. Localization was observed under a confocal laser scanning microscope (LSCM) with an excitation peak of 395 nm and an emission peak of 509 nm.

### Analysis of transcriptional activation activity of tea plant CsBES1-14 protein

The transcriptional activity of *CsBES1-14* was assessed using segmented constructs: the full-length protein (528 bp), an N-terminal fragment (corresponding to nucleotides 1–264 bp), and a C-terminal fragment (spanning nucleotides 265–528 bp). Homologous recombination was employed to create the yeast expression vectors pGBKT7-CsBES1-14 (full-length), pGBKT7-CsBES1-14 (N-terminal), and pGBKT7-CsBES1-14 (C-terminal). The preparation of yeast competent cells adhered to the guidelines provided by the Coolaber Classic Yeast Transformation Kit. The recombinant plasmids—pGBKT7-CsBES1-14 (full-length), pGBKT7-CsBES1-14 (N-terminal), and pGBKT7-CsBES1-14 (C-terminal)—along with both positive and negative controls, were introduced into the Y2HGold yeast competent cells that had been set up. Yeast cultures were diluted and plated post-transformation on synthetic complete medium amended with 30 mg/ml X-α-gal. After 2–3 days of incubation at 30°C, phenotypic observations (growth and color) were made for experimental and control sets (Yeasen, Shanghai, China; Cat. 10923ES05).

### Plant growth and stress treatment

The tea plant material used for cold stress treatment in this study was ‘Qiancha 1’, all of which were 1-year-old tea seedlings in good growth condition for the experiment. This variety was planted in the tea garden of Guizhou University, China. Tea plant can be propagated by cuttings, and tea branches are widely used as experimental materials. The growth conditions and treatment conditions of tea plant in the BL-spraying and cold treatment experiments refer to Yang [55]. Meanwhile, the working concentration of the BR inhibitor BRZ was 10 μM, and the sampling time was consistent with the sampling time after BL treatment. It was used to evaluate the expression of *CsBES1-14* and *CsCOR413*. Each experimental group included three replicates.

To verify the function of CsBES1-14, we selected WT (Col) and loss-of-function mutant *A. thaliana* (*bes1-1*) for overexpression analysis. The transgenic *A. thaliana* and WT *A. thaliana* seeds were sterilized and cold-treated at 4°C for 2 days, then sown on one-half MS medium. Uniformly growing T3 generation pure transgenic lines (Col/*CsBES1-14*-OE, *bes1-1*/*CsBES1-14*-OE) and control plants (Col, *bes1-1*) were selected. Three biological replicates were established for each genotype, with each replicate containing 12 two-week-old *A. thaliana* plants. The culture conditions were in accordance with Yang [55].

### Isolation of RNA followed by RT-qPCR analysis

Total RNA from young *C. sinensis* leaves was purified using a phenolic-compound-optimized isolation system (Huayueyang Biotech Co. Ltd, Beijing, China). Subsequent first-strand cDNA synthesis employed the Prime Script RT system (Takara, Beijing, China). The measurement of gene expression levels was facilitated by the NovoStart® SYBR qPCR Super Mix plus (Novoprotein, Shanghai, China, E096) in conjunction with the CFX Connect™ system (Bio-Rad, California, USA). The internal reference genes utilized included *CsGAPDH* and *Atactin2/8*, with primers for the genes designed utilizing Primer 5.0 (Table S1). The 2^−ΔΔCt^ method was applied to determine relative expression of all target genes. Data normality was verified by Shapiro–Wilk testing, with variance homogeneity confirmed via Levene’s method. Group comparisons were made using one-way analysis of variance (ANOVA), provided that the assumptions of normal distribution and variance homogeneity were met.

### Observation of cold-tolerance phenotype in transgenic plants

Plants that were cultivated under standard conditions underwent a low-temperature treatment using a light incubator set to low temperatures. The chilling regimen initiated at 0°C and decreased 2°C bihourly until achieving the target temperature, which remained stable for 2 h. After this period, the culture dishes were taken out and promptly transferred to a dark environment at 4°C for overnight storage. Once the plants returned to normal growth conditions for ~2–3 days, the surviving plants were tallied, and the survival rate was computed.

### Physiological index measurement

#### Relative conductivity measurement

The analysis was conducted following the method outlined by Li *et al*. [18]. Following the freezing experiment conducted with *A. thaliana*, the seedlings were placed into a test tube filled with 8 ml of distilled water, and the initial conductivity was noted as S_0_. Following 15-min incubation at 22°C with shaking, conductivity was measured and designated S₁. Subsequently, the samples underwent boiling for 20 min and were shaken again at 22°C for another 20 min, after which a final conductivity measurement was taken, resulting in S_2_. Electrolyte leakage rate was derived as (S₁ − S₀)/(S₂ − S₀) × 100%.

#### DAB and NBT histochemical staining

Leaf samples were subjected to immersion in: a DAB solution (1 mg/ml, pH 5.8) and an NBT solution (0.2%, pH 7.5). The samples were then maintained at 28°C in darkness for a period of 12 h. Following this incubation phase, the samples were immersed in anhydrous ethanol for a duration of 24 h, and subsequently, they were boiled in water at 100°C for 5 min to facilitate decolorization. Once cooled to room temperature, photographs of the samples were captured.

### Silencing of tea plant *CsBES1-14* gene using VIGS technology

Experiments on VIGS in tea plant were performed using the TRV vector. A fragment measuring 279 bp, specifically from the 10th to the 289th base pair of the *CsBES1-14* gene, was utilized to create the VIGS vector. Branches from tea plant that had grown to a height of 10 cm were chosen; the apical buds were removed while retaining one to two healthy leaves. These branches were submerged in a resuspended solution for 20 min, then subjected to vacuum infiltration at a pressure of 0.7 KPa for 4 min, with this process repeated once more. After inoculation, branches were hydroponically cultivated in Hoagland solution: first under 24°C dark conditions for 4 days, then transferred to a photoperiod regime (16 h light/8 h dark) at constant temperature for continued growth. For comparison, the negative control used was the empty pTRV2 vector, while the positive control was designated as TRV-*CsPDS*.

### Measurement of relative conductivity in tea plant after cold treatment

Approximately 0.1 g of midrib-removed tea leaves from control and treated groups were transferred to sterile tubes containing 10 ml deionized water. The samples were subjected to vacuum treatment for half an hour, then agitated at room temperature for 1 h, after which their conductivity was recorded as S_1_. After boiling all samples for 20 min and cooling to room temperature, post-treatment conductivity (S₂) was measured. Relative conductivity was calculated as (S₁/S₂) × 100%.

### Identification of downstream target genes of *CsBES1-14* using transcription factor-centered Y1H screening.

Since *CsBES1-14* exhibits transcriptional activation activity, the prey vector pGADT7-*CsBES1-14* was transformed into Y187 yeast competent cells. The transformed cells were plated on SD/-Trp/-Leu medium containing different concentrations of 3-amino-1,2,4-triazole (3AT) for screening. At a concentration of 100 mM 3AT, the transcriptional activity was suppressed. Subsequently, the bait vector pGADT7-*CsBES1-14* was cotransformed with a prey motif library into Y187 yeast competent cells. The transformed culture was plated on selective medium lacking Trp, Leu, and His and supplemented with 100 mM 3AT for screening (Fig. S10a). A total of 40 yeast clones were obtained and subjected to colony PCR validation followed by DNA sequencing. After sequence alignment, 40 distinct motifs were identified. Analysis of these motifs revealed that GTCTGTT, CACGAAC, GTTGTTG, GAAACTA, and AAAACGT contain *cis*-acting elements responsive to low temperature, MeJA, and ABA (Fig. S10b and c). These five motifs were used to scan the tea plant genome, leading to the identification of *CsCOR413* (TEA000894.1), a cold-related protein.

### Yeast one-hybrid reverse verification

Using homologous recombination, we cloned the full-length CDS of *CsBES1-14* into the pGADT7 (AD) prey vector in a single step. Simultaneously, a 2000-bp promoter fragment upstream of the *CsCOR413* gene was cloned into the pHIS2 vector. Subsequent to this, yeast reverse verification was performed in accordance with the guidelines provided by the traditional yeast transformation kit (SK2400, Coolaber).

### Dual luciferase assay verification

To construct the dual-luciferase vectors, the *CsBES1-14* gene fragment was cloned into the pGreenII 62-SK vector, and a 2000-bp promoter region of the *CsCOR413* gene was inserted into the pGreenII 0800-Luc vector using a one-step cloning method. Following this, the plasmids were introduced into Agrobacterium EHA105 competent cells. The Agrobacterium containing the target gene plasmids was streaked on YEP plates enriched with 20 mg/ml Rifampicin (Rif) and 100 mg/ml Kanamycin (Kan), and then incubated at 28°C for a period of 2–3 days. Individual colonies were picked and transferred to 1 ml of YEP medium amended with 20 mg/ml Rif and 100 mg/ml Kan. These cultures were then shaken (200 rpm) at 28°C for 48–72 h. Subsequently, 0.5 ml of this bacterial culture was transferred to 50 ml of YEP medium and incubated at 28°C with shaking at 200 r/min until the optical density at 600 nm (OD_600_) reached between 0.6 and 0.8. The bacterial cells were then centrifuged, the resulting pellet was resuspended, and this resuspension was mixed in the correct proportions. After thorough mixing, the bacterial suspension was injected into four small openings made in each leaf. The leaves were cultured in darkness overnight, and then placed under normal light for an additional 2 days. Under standard experimental conditions, a solution of 0.2 mg/ml D-luciferin sodium salt was introduced for a incubation period of 5–10 min, followed by observation and image acquisition with a luminometer.

### Electrophoretic mobility shift assay

Following cloning into the PGEX-4 T-GST vector, the CsBES1-14 fusion protein was expressed and subsequently purified. The 2000-bp promoter region upstream of the *CsCOR413* gene was analyzed using the online prediction tools JASPAR (https://jaspar.elixir.no/) and MEME (https://meme-suite.org/meme/tools/meme), and six potential transcription factor-binding sites were identified. Based on these sites, double-stranded oligonucleotide probes containing the predicted binding motifs were designed and synthesized. These probes were then tagged with either biotin or radioactive isotopes. The labeled probes were combined with the purified protein and allowed to incubate at room temperature to establish protein–probe complexes. To differentiate the protein–probe complexes from the unbound probes, a nondenaturing polyacrylamide gel electrophoresis was conducted. Proteins were transferred from the gel onto a PVDF membrane, followed by detection via streptavidin-HRP and chemiluminescent substrate.

### Physiological response assay

Physiological parameters, including Pro content and the activities of POD, CAT, and SOD, were measured in leaves from different plant species. The assays were performed using specific detection kits for Pro, POD, CAT, and SOD (Boxbio, Beijing, China; Cat. No. AKAM003M, AKAO005C, AKAO003-1U, and AKAO001M, respectively), following the manufacturer’s protocols.

### Statistical analysis

All experiments were performed with at least three independent biological replicates. Each physiological parameter was expressed as mean ± SE. The normality of the data was verified using the Shapiro–Wilk test, and the homogeneity of variance was confirmed by Levene’s test. For data meeting the assumptions of normality and homogeneity of variance, between-group comparisons were conducted using one-way or two-way ANOVA, with a significance level of α = 0.05, followed by the Tukey HSD test for *post hoc* multiple comparison corrections. Bar graphs were generated using GraphPad Prism 10.0.

## Supplementary Material

Web_Material_uhag098

## Data Availability

All data supporting the findings of the study are available.
