# Supplementary material for: The CsBES1-14-CsCOR413 module mediated by brassinolide positively regulates cold resistance in tea plant
Source: Hortic Res. 2026 Mar 13;13(7):uhag098. doi: 10.1093/hr/uhag098 (PMC13278839; doi:10.1093/hr/uhag098)
Supplement: Web_Material_uhag098 [file web_material_uhag098.pdf]

	Primer sequence
CsGAPDH	F: CACGGTCAATGGAAGCATCAT
	R: GCAGCAGCCTTATCCTTATCAG
CsBES1-14（RT）	F: AGGAGTGCCATGAGAGGAT
	R: CGTCTTCTTCGCTCCCTATT
GUS（RT）	F: ACTGCTGCTGTCGGCTTTA
	R: GCACTTGCGGACGGGTAT
AtACTIN（RT）	F: GGTAACATTGTGCTCAGTGGTGG
	R: AACGACCTTAATCTTCATGCTGC
AtCOR15A(RT)	F: ACTCAGTTCGTCGTCGTTTCTC
	R: CTCACCATCTGCTAATGCCTCT
AtCOR47(RT)	F: CAGTGTCGGAGAGTGTGGTG
	R: ACAGCTGGTGAATCCTCTGC
CsSK1(RT)	F: CAGCGTGTTGTTGGGACTG
	R: CGGTTCTTGTATCTTCTGTCTTG
CsCOR47(RT)	F: GAGCGATGAGGAGTATGAAGAC
	R: TGTCACCACTGCCTCGTATT
CsCOR413(RT)	F: CATGTGCAGATAGTTCTATG
	R: GTCGTCTTCATTGCCAAATA
CsTCH4(RT)	F: AGCTTGTCCCTGGAAACTCTG
	R: CTCTCTGTTGCCTTTGCCTTG
CsICE1(RT)	F: CGCAGACTTGAACCACCCT
	R: TCTGGGATGGTTTGGAAGT
CsCBF1(RT)	F: TTGTTGTGAGGGTTAGTTGC
	R: CTGACGATAAAGGCTGTAATG
CsCYCD3(RT)	F: GGGAAGACGAAGAGTTGACCT
	R: CCGTGAGAGCAGAGAATGAGT

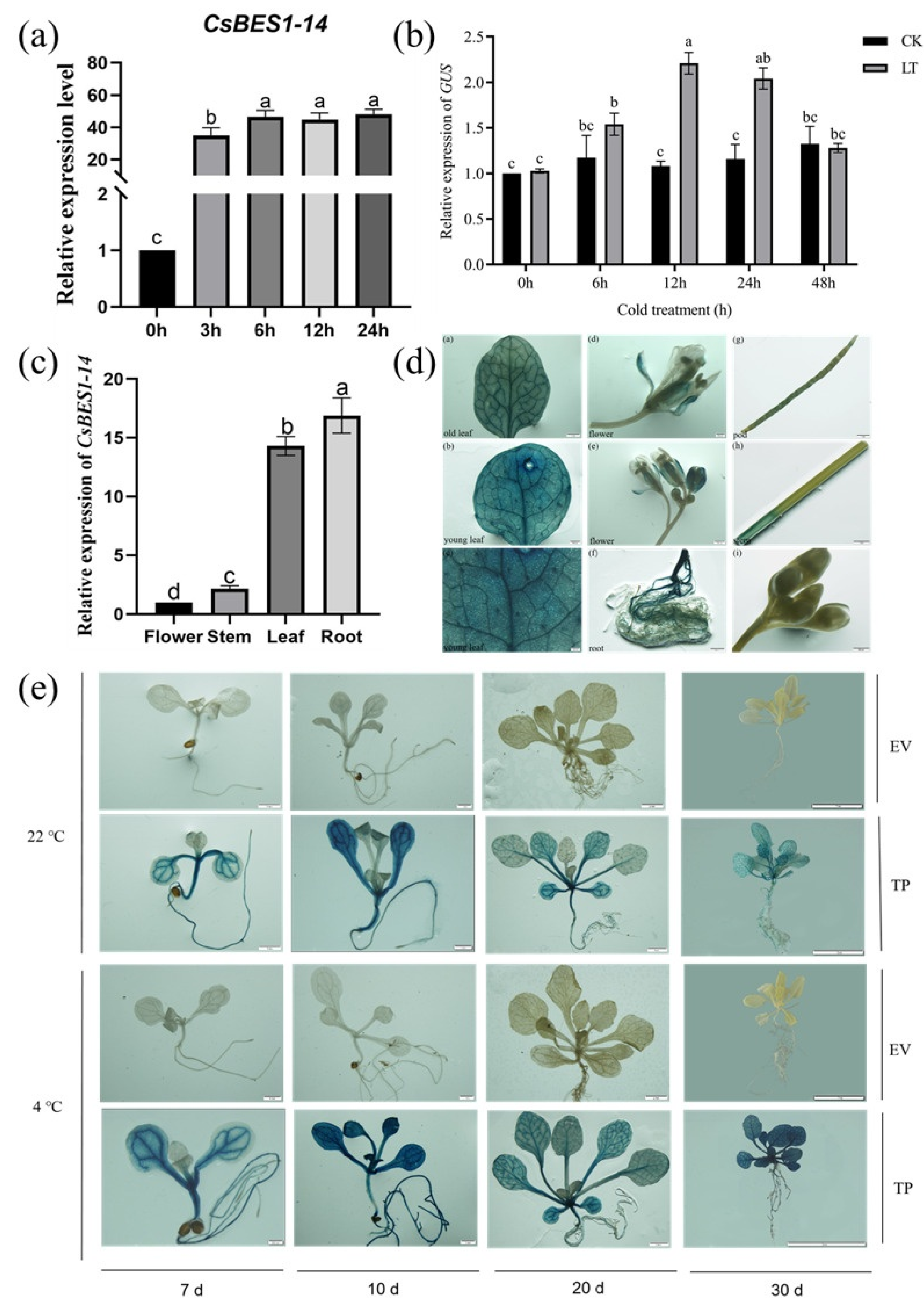

(a)
CsBES1-14
Relative expression level
80
60
40
20
2
1
0
c
b
a
a
a
0h
3h
6h
12h
24h
(b)
CK
LT
Relative expression of GUS
2.5
2.0
1.5
1.0
0.5
0.0
c
c
bc
b
c
a
c
ab
bc
bc
0h
6h
12h
24h
48h
Cold treatment (h)
(c)
Relative expression of CsBES1-14
20
15
10
5
0
d
c
b
a
Flower
Stem
Leaf
Root
(d)
(a)
old leaf
(d)
flower
(g)
pod
(b)
young leaf
(e)
flower
(h)
(f)
root
(i)
(e)
22 °C
4 °C
EV
TP
EV
TP
7 d
10 d
20 d
30 d

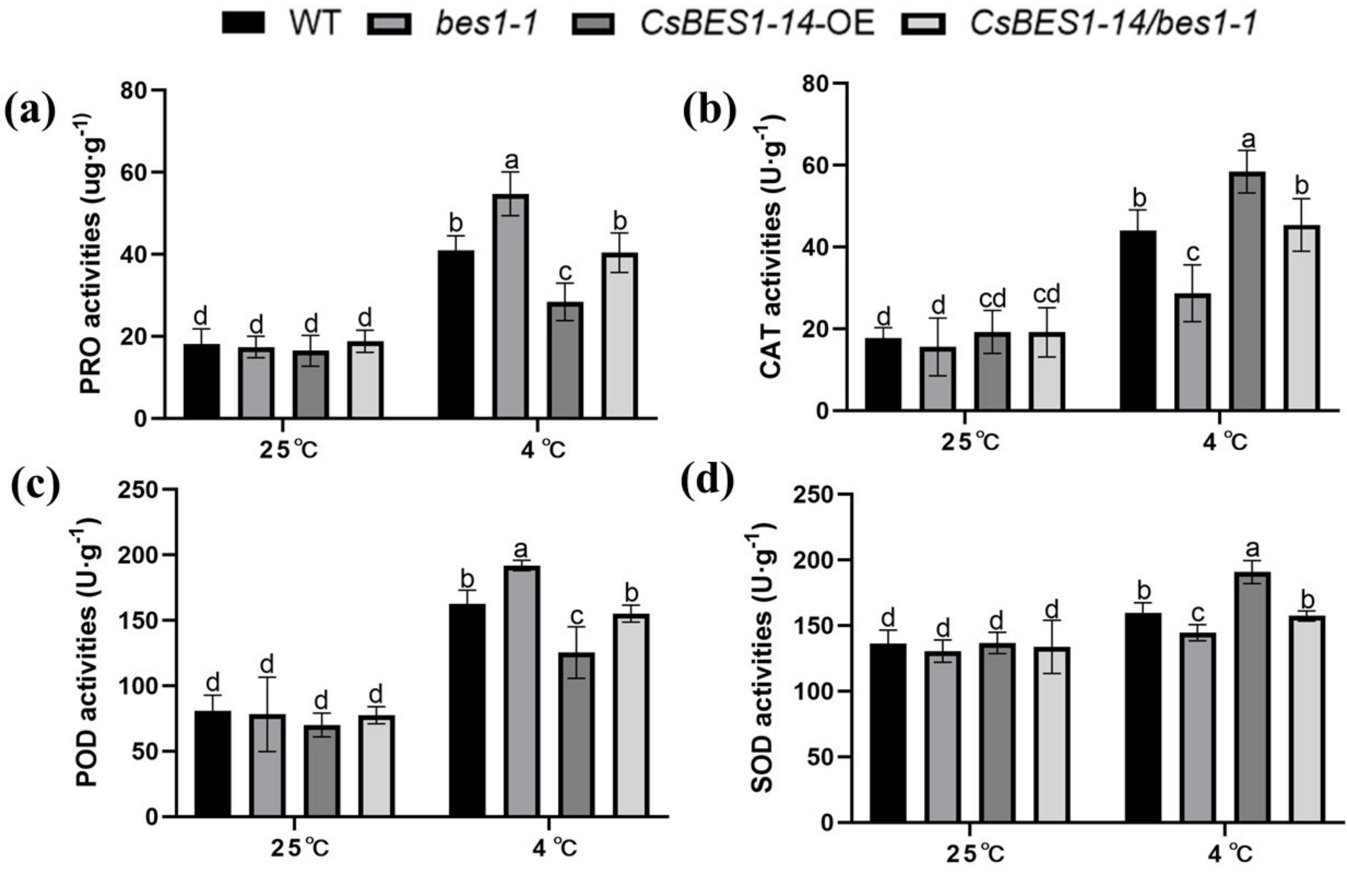

WT
bes1-1
CsBES1-14-OE
CsBES1-14/bes1-1
(a)
PRO activities (ug·g-1)
(b)
CAT activities (U·g-1)
(c)
POD activities (U·g-1)
(d)
SOD activities (U·g-1)
25℃
4℃

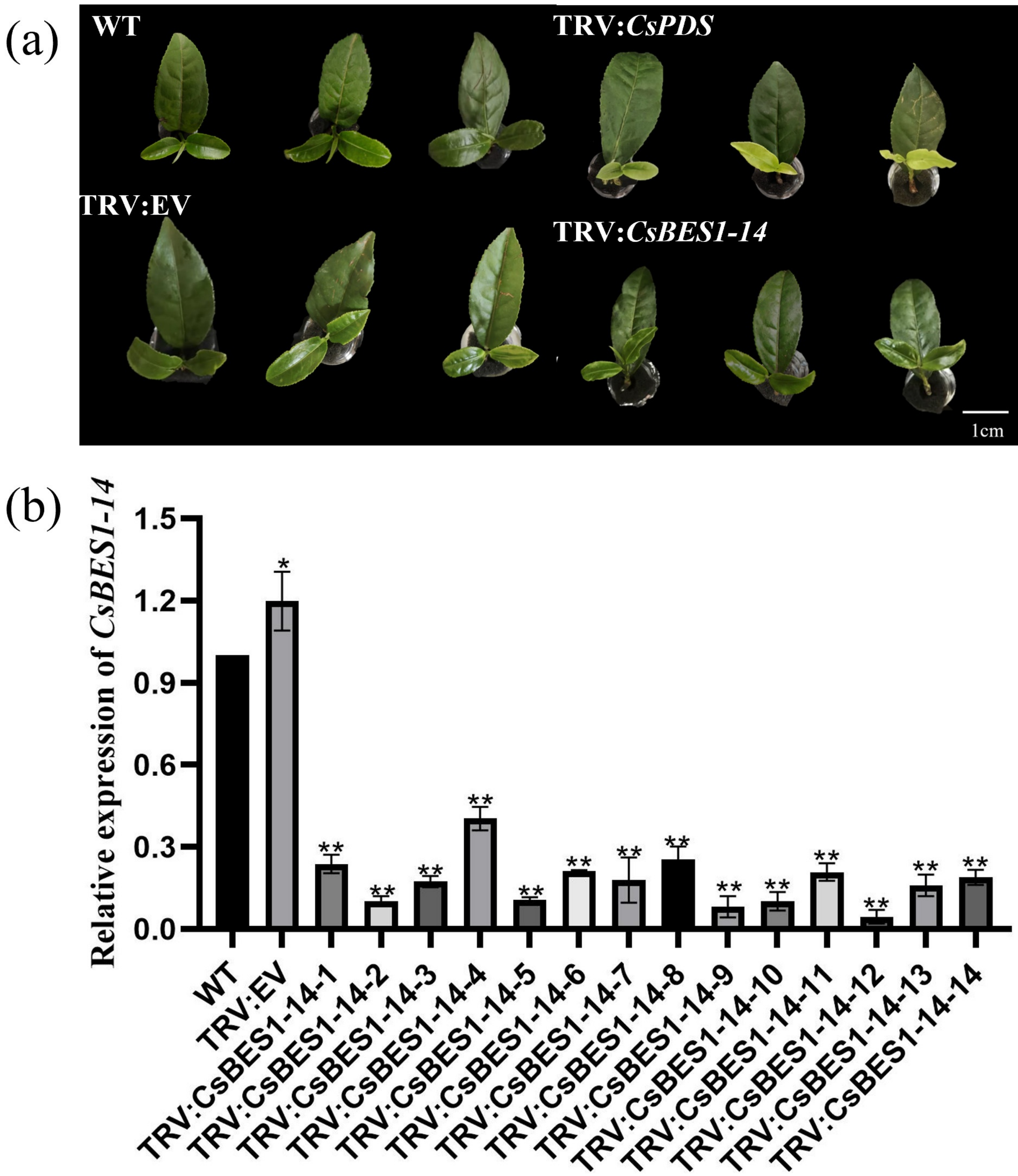

(a)
WT
TRV:*CsPDS*
TRV:EV
TRV:*CsBES1-14*
1cm
(b)
Relative expression of *CsBES1-14*
1.5
1.2
0.9
0.6
0.3
0.0
*
**
WT
TRV:EV
TRV:CsBES1-14-1
TRV:CsBES1-14-2
TRV:CsBES1-14-3
TRV:CsBES1-14-4
TRV:CsBES1-14-5
TRV:CsBES1-14-6
TRV:CsBES1-14-7
TRV:CsBES1-14-8
TRV:CsBES1-14-9
TRV:CsBES1-14-10
TRV:CsBES1-14-11
TRV:CsBES1-14-12
TRV:CsBES1-14-13
TRV:CsBES1-14-14

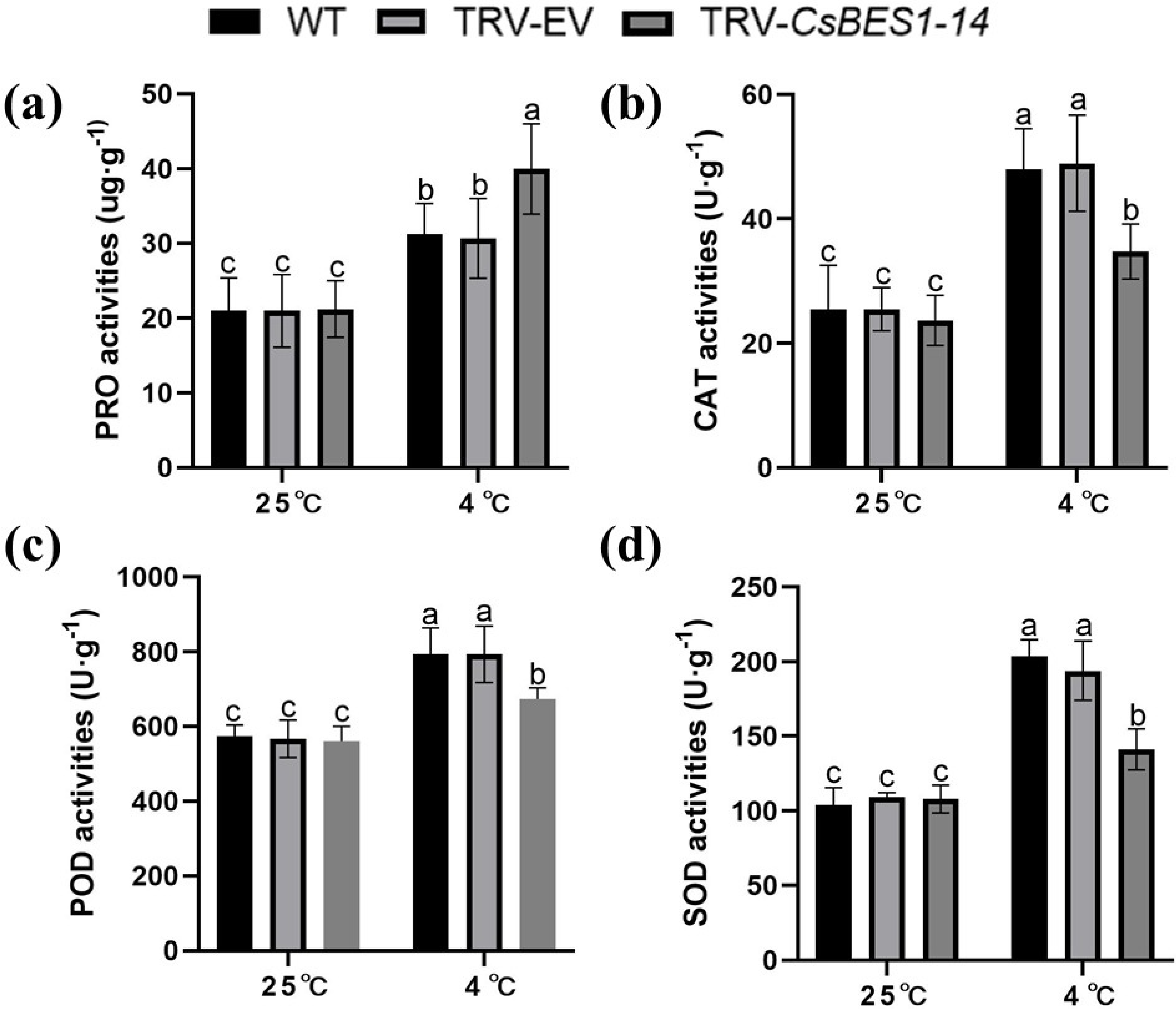

WT
TRV-EV
TRV-CsBES1-14
(a)
PRO activities (ug·g-1)
25℃
4℃
(b)
CAT activities (U·g-1)
25℃
4℃
(c)
POD activities (U·g-1)
25℃
4℃
(d)
SOD activities (U·g-1)
25℃
4℃

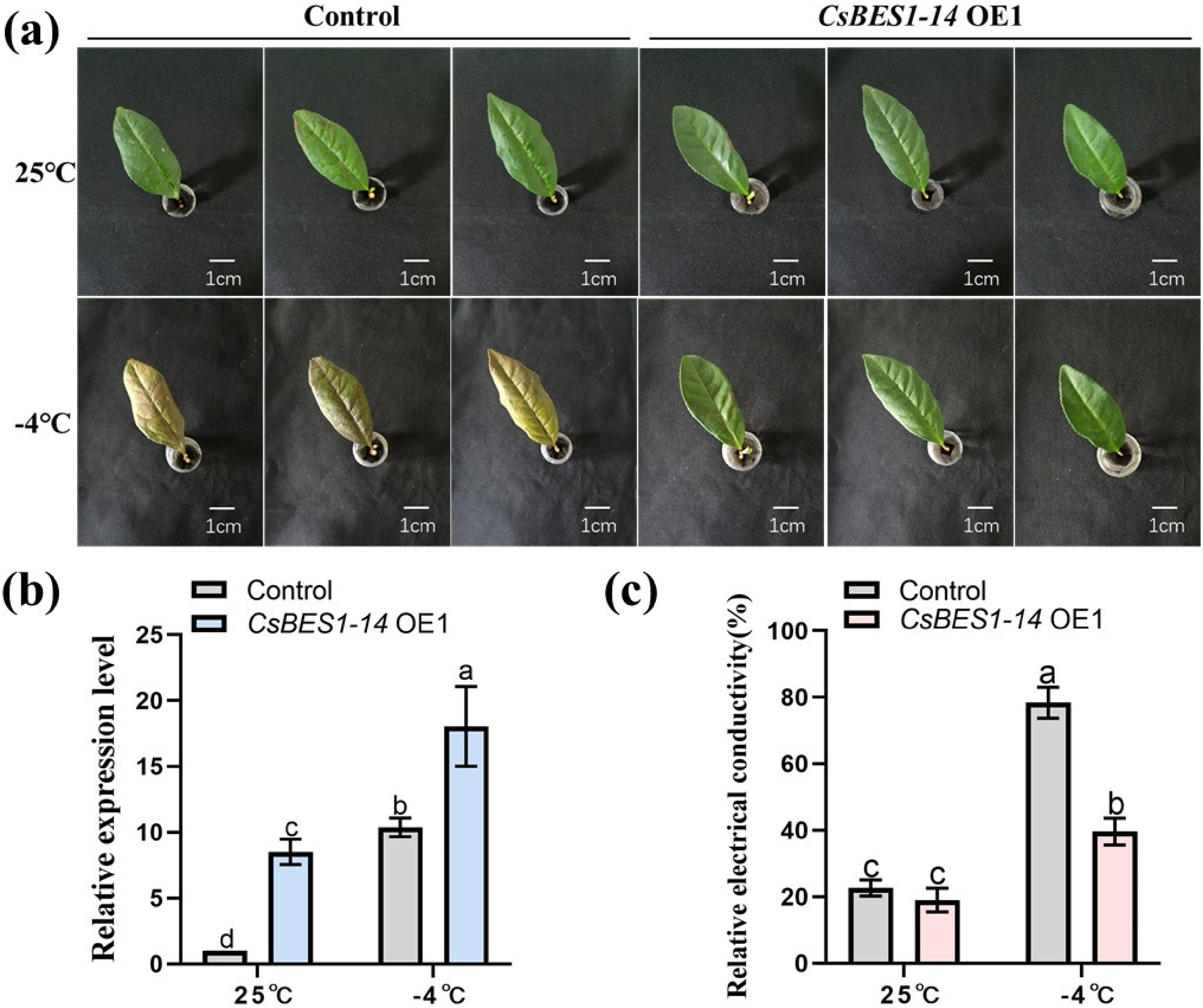

(a)
Control
CsBES1-14 OE1
25°C
-4°C
1cm
(b)
Control
CsBES1-14 OE1
Relative expression level
25
20
15
10
5
0
d
c
b
a
25℃
-4℃
(c)
Control
CsBES1-14 OE1
Relative electrical conductivity(%)
100
80
60
40
20
0
c
c
a
b
25℃
-4℃

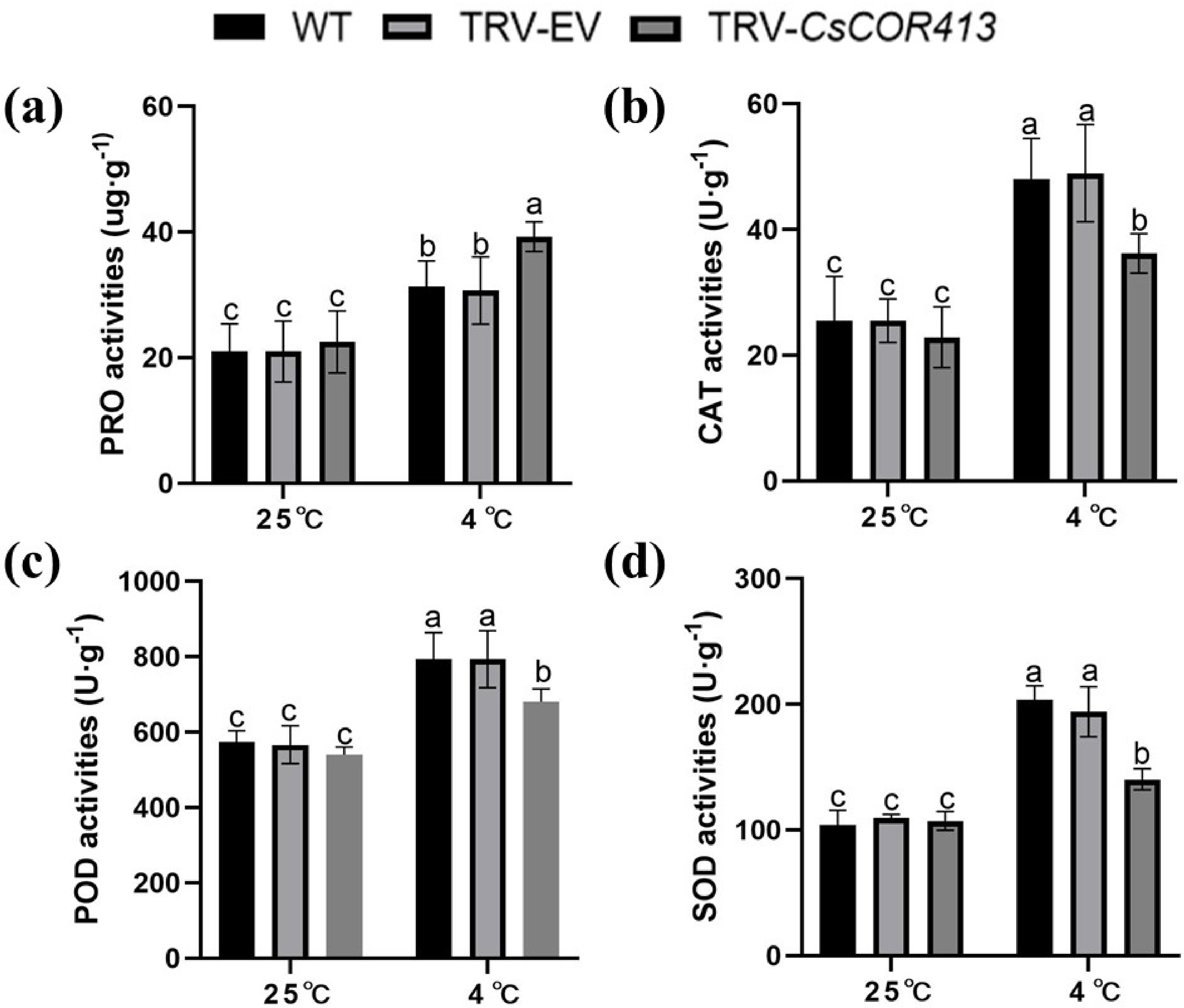

WT
TRV-EV
TRV-CsCOR413
(a)
PRO activities (ug·g-1)
60
40
20
0
25℃
4℃
(b)
CAT activities (U·g-1)
(c)
POD activities (U·g-1)
1000
800
600
400
200
(d)
SOD activities (U·g-1)
300
200
100

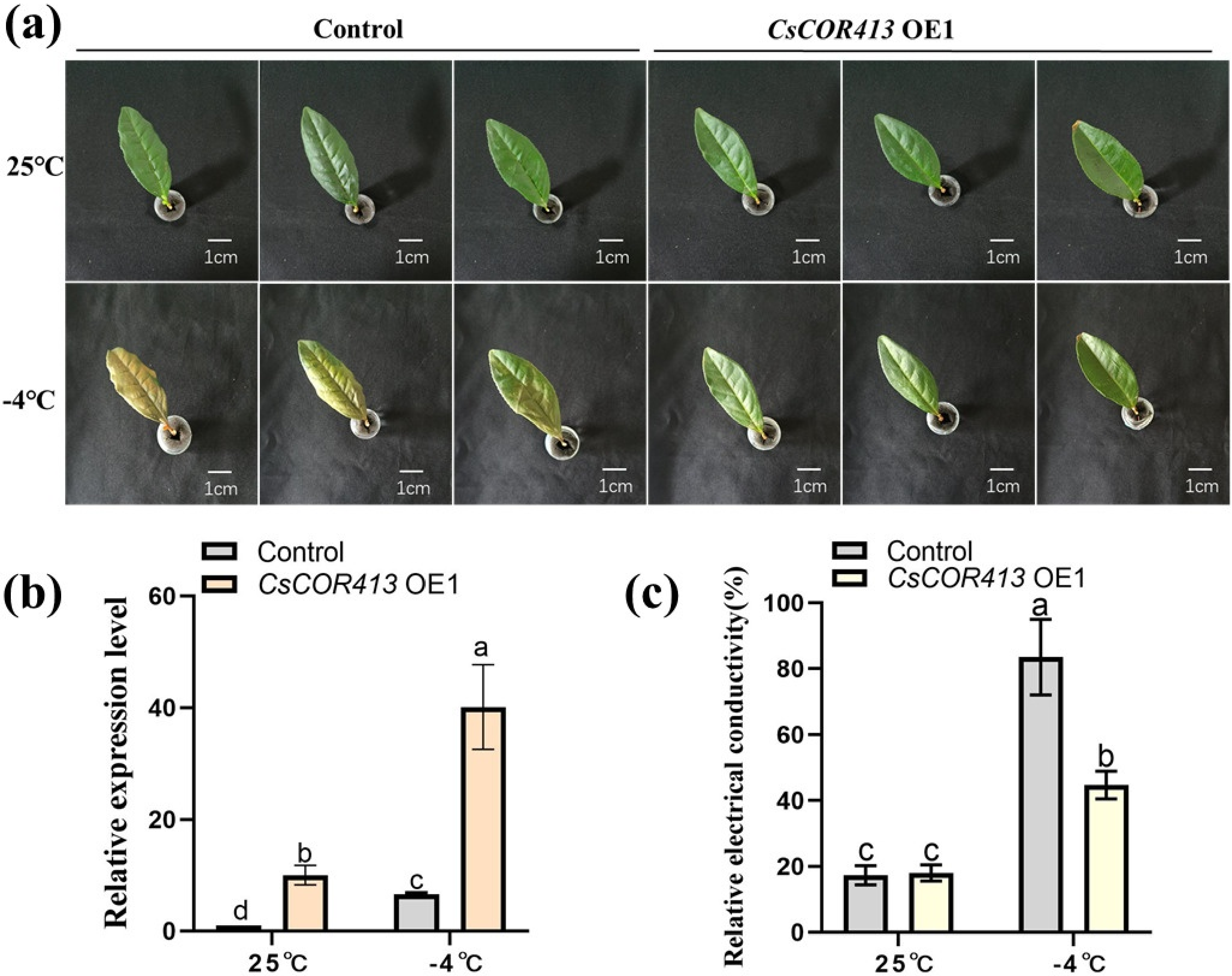

(a)
Control
CsCOR413 OE1
25℃
-4℃
1cm
(b)
Control
CsCOR413 OE1
Relative expression level
60
40
20
0
d
b
c
a
25℃
-4℃
(c)
Control
CsCOR413 OE1
Relative electrical conductivity(%)
100
80
60
40
20
0
c
c
a
b
25℃
-4℃

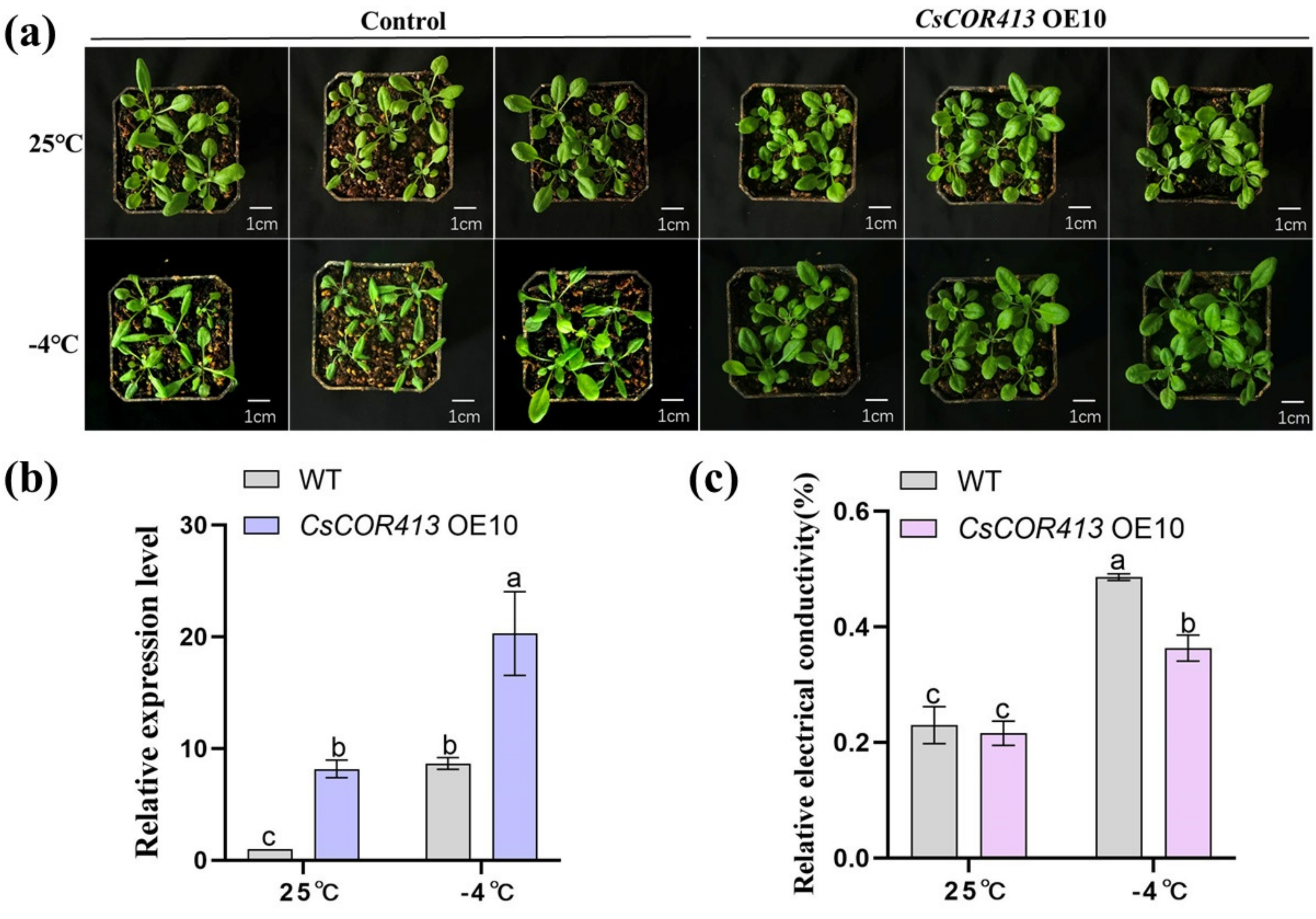

(a)
Control
CsCOR413 OE10
25°C
-4°C
1cm
(b)
WT
CsCOR413 OE10
Relative expression level
30
20
10
0
25℃
-4℃
a
b
b
c
(c)
WT
CsCOR413 OE10
Relative electrical conductivity(%)
0.6
0.4
0.2
0.0
25℃
-4℃
a
b
c
c

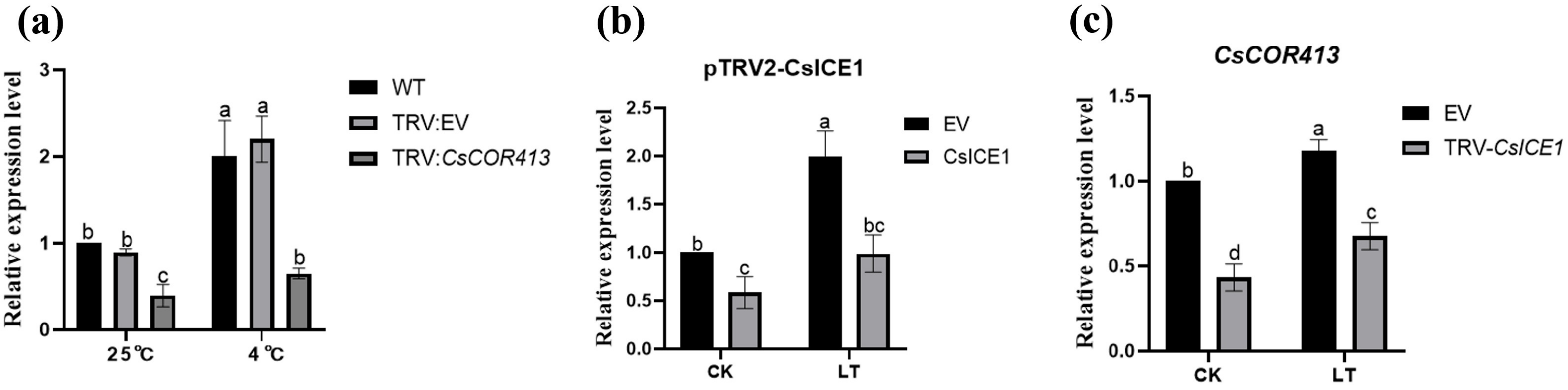

(a)
Relative expression level
WT
TRV:EV
TRV:CsCOR413
25℃
4℃
(b)
pTRV2-CsICE1
Relative expression level
EV
CsICE1
CK
LT
(c)
CsCOR413
Relative expression level
EV
TRV-CsICE1
CK
LT

(a)

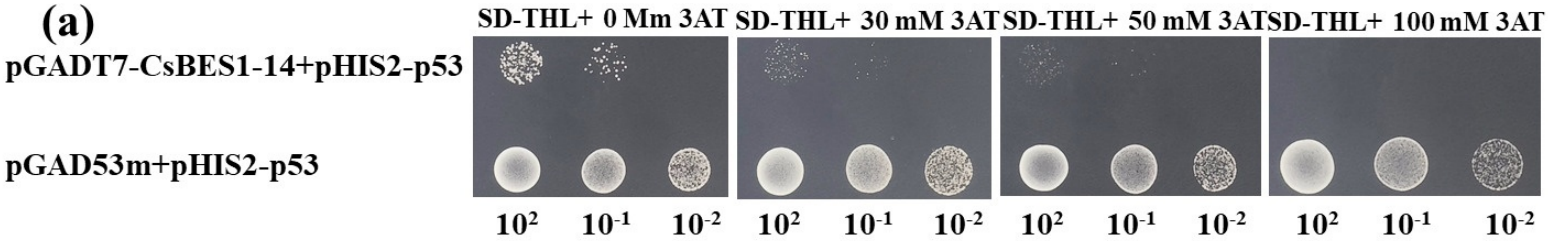

(b) SD-TLH + 100 mM 3AT

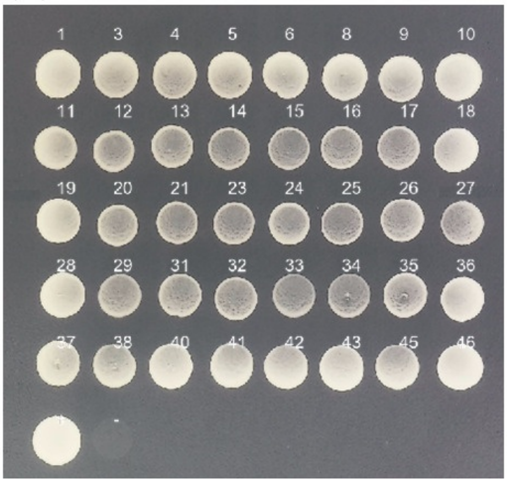

(c)

CCCGCGC	CTAAAGA	GTCTGTT	GTGCAAG	GTGCCTA
GACGAGG	CACGAAC	ACTGCGG	GGTGGCT	CGCATGG
ATCGGCC	ACCGCTG	GTCGGTC	GACCGAA	GAAGGTA
TTTGCTC	CCGCAGT	CTTTACA	GTTGTGG	TAGCAAA
GACAAAC	GCGGCAC	GTGCCTC	AGGTATC	CGCAAGC
CCCGCGA	GAAACTA	ACAATAG	AATGATC	CAGAGCC
GAGGAAC	GCGCTTG	GTCGCTG	GTATTTC	GCCGGAT
TACGGTA	AAAACGT	GAACGAG	CTGTAGT	AGGAGGG
